# Pediatric Brain Tissue Segmentation Using a Snapshot Hyperspectral Imaging (sHSI) Camera and Machine Learning Classifier

**DOI:** 10.3390/bioengineering10101190

**Published:** 2023-10-13

**Authors:** Naomi Kifle, Saige Teti, Bo Ning, Daniel A. Donoho, Itai Katz, Robert Keating, Richard Jaepyeong Cha

**Affiliations:** 1 Sheikh Zayed Institute for Pediatric Surgical Innovation, Children’s National Hospital, Washington, DC 20010, USA; nkifle@childrensnational.org (N.K.); itai.j.katz@gmail.com (I.K.); 2 Department of Neurosurgery, Children’s National Hospital, Washington, DC 20010, USA; sateti@childrensnational.org (S.T.); ddonoho@childrensnational.org (D.A.D.); 3 Department of Pediatrics, George Washington University School of Medicine, Washington, DC 20010, USA

**Keywords:** pediatric brain tumor, neurosurgery, snapshot hyperspectral imaging, random forest, segmentation

## Abstract

Pediatric brain tumors are the second most common type of cancer, accounting for one in four childhood cancer types. Brain tumor resection surgery remains the most common treatment option for brain cancer. While assessing tumor margins intraoperatively, surgeons must send tissue samples for biopsy, which can be time-consuming and not always accurate or helpful. Snapshot hyperspectral imaging (sHSI) cameras can capture scenes beyond the human visual spectrum and provide real-time guidance where we aim to segment healthy brain tissues from lesions on pediatric patients undergoing brain tumor resection. With the institutional research board approval, Pro00011028, 139 red-green-blue (RGB), 279 visible, and 85 infrared sHSI data were collected from four subjects with the system integrated into an operating microscope. A random forest classifier was used for data analysis. The RGB, infrared sHSI, and visible sHSI models achieved average intersection of unions (IoUs) of 0.76, 0.59, and 0.57, respectively, while the tumor segmentation achieved a specificity of 0.996, followed by the infrared HSI and visible HSI models at 0.93 and 0.91, respectively. Despite the small dataset considering pediatric cases, our research leveraged sHSI technology and successfully segmented healthy brain tissues from lesions with a high specificity during pediatric brain tumor resection procedures.

## 1. Introduction

Brain tumors are the most common solid tumors in children and account for the highest number of cancer-related deaths worldwide [1]. The main symptoms include headaches, seizures, nausea, drowsiness, and microcephaly. When left untreated, they can lead to coma or death. While developing a treatment plan, neurosurgeons need to understand the tumor type, grade, category, and location. There are two types of tumors: benign and malignant [2]. Benign tumors grow slowly and are non-cancerous, whereas malignant tumors are extremely aggressive. The grade refers to the level of aggressiveness of the tumor cells; a higher grade indicates more malignant tumors [2]. Considering that tumors may transform into higher grades, early treatment is critical. Therefore, it is vital to know their categories, which are primary and secondary (or metastatic). Primary tumors originate in the brain, whereas metastatic tumors originate in other parts of the body and move up to the brain [2]. Finally, the tumor location is determined by scanning the brain (via CTs or MRIs) [3], which is important for assessing the type of surgery required for removal or biopsy. 

The current intraoperative standard for surgeons to evaluate tumor margins is to send small pieces of tissue for biopsy to the pathology department for testing, which is time-consuming and increases the duration of surgery, as the tests do not provide real-time results and are often not definitive. This usually requires the surgeon to make real-time decisions based upon the appearance of the different tissues including what is likely a tumor versus normal brain tissue. Often, tumors, especially low-grade tumors, appear indistinguishable from normal brain tissue. Snapshot hyperspectral imaging (HSI) can potentially identify tumor margins in real time and thus can be used to help or contribute to a greater degree of tumor resection as well as minimize morbidity. In addition, this can be used to teach less-experienced surgeons to identify tumors from normal brain tissue [4].

HSI is an imaging technique that captures and processes a wide spectrum of light between visible and infrared wavelengths. Unlike the conventional RGB image, which uses only three colors (red, green, and blue), HSI captures spectral data at each pixel of the image. These data are then used to create a three-dimensional hyperspectral data cube containing spatial and spectral information [5]. The wide range of spectral information allows HSI to identify different materials and objects with unique high-resolution spectral properties [6,7]. Different materials exhibit different light reflection, absorption, and scattering responses [8]. Although HSI was originally developed for remote sensing [9,10], it is widely used in medical imaging owing to its noninvasive imaging modality, which helps collect spatial and spectral information from tissues [6].

Some of the common uses of HSI in medical imaging include identifying different types of tissues and cancers [11,12,13], monitoring treatment responses, and providing [6] surgical guidance [14]. This is possible because HSI provides detailed information on tissue properties and biochemical processes that cannot be visualized using traditional imaging or visible wavelengths. More specifically, in cancer cases, HSI can detect biochemical and morphological changes in tissues, which aids in diagnosis [14]. Additionally, HSI spectral signatures help extract and differentiate cancerous and normal tissues [13]. 

Furthermore, HSI can be used intraoperatively for real-time guidance in identifying tumor margins and achieving more accurate and complete tumor removal [13]. Significant advances have been made in machine learning-based tumor diagnosis. In a study by Shokouhifar et al. [15], they used a three-stage deep learning ensemble model embedded in a camera scanning tool to measure the volume of the arm of patients with lymphedema. The model was very successful, allowing for patients to be measured in an inexpensive and noninvasive manner. Another used of machine learning for tumor detection is classification. Veeraiah et al. [16] used the mayfly optimization with a generative adversarial network to classify different types of leukemia from blood smear images. Other innovations in machine learning-based tumor segmentation have also been achieved [17,18], including brain tumor segmentation using machine learning [19,20,21,22]. Kalaivani et al. [23] used machine learning to segment brain tumors based on MRI images. The collected MRI images were pre-processed through denoising to remove irrelevant information and improve image quality. Features were extracted, and three machine learning classifiers, i.e., Fuzzy C-Mean Clustering (FCM), K-nearest neighbor (KNN), and K-means, were implemented to classify the areas of the MRI images as tumor or nontumor regions. The classifiers were highly successful with segmentation accuracies of 98.97%, 89.96%, and 79.95% for FCM, KNN, and K-means, respectively. Combining both segmentation and classification, Eder at al. were able to use segmented MRI images of patients with brain tumors to predict if the patient would survive [24].

By combining HSI and machine learning classifiers, Ruiz et al. [25] classified the regions of the HSI images of four patients with glioblastoma grade IV brain tumors. This classification was performed using random forest and support vector machine (SVM), and the goal was to train the models to classify the regions of the image into five classes: healthy tissue, tumor, venous blood vessel, arterial blood vessel, and dura mater. Two experiments were conducted: the first experiment considered 80% of the images of each patient for training and the remaining 20% for testing, and the second experiment considered three patients for training and one for testing. In the first experiment, random forest slightly outperformed SVM with almost-perfect accuracy scores. However, in the second experiment, SVM exhibited significantly better accuracy. 

In another study, Ma et al. [26] used a hyperspectral microscopic imaging system to detect head and neck cancer nuclei on histological slides. The HSI and co-registered RGB images were trained using a convolutional neural network (CNN) for nuclear classification. Compared with the RGB CNN, which had a test accuracy of 0.74, the HSI CNN produced significantly better results, with a test accuracy of 0.89 because the RGB CNN uses spatial information, whereas the HSI CNN uses spatial and spectral information.

Similar to earlier studies, we introduce a new snapshot hyperspectral camera with a random forest classifier. Snapshot HSI helps capture hyperspectral images in a single exposure. Although this technique is extensively used in astronomy [27], it is rarely used in the medical domain. The snapshot HSI sensor is ideal for medical imaging because it is noninvasive and nonionizing; however, it acquires large datasets in real time. Currently, there is an unmet need to examine anatomical structures beyond the visible human spectrum, specifically in a manner that is unobtrusive to the surgical workflow. Although brain tumors have clear margins and are easily excised, others are diffused or located in critical brain structures. Using our HSI device, we can differentiate tissue types by observing their characteristic spectra and training a deep learning classifier, such as a random forest, to perform pixel-level segmentation. 

**Key Achievements:**

- We developed a compact sHSI camera designed for seamless integration with an existing surgical microscope, enabling remote control for the simultaneous acquisition of both color and hyperspectral data.
- Our study harnessed sHSI technology to capture real-time images extending beyond the visible spectrum, effectively distinguishing healthy brain tissues from lesions in surgical scenarios.
- We conducted machine learning model training by utilizing data from pediatric patients and assessed the resulting performance outcomes.

## 2. Materials and Methods

### 2.1. Data Collection

**Figure 1:**
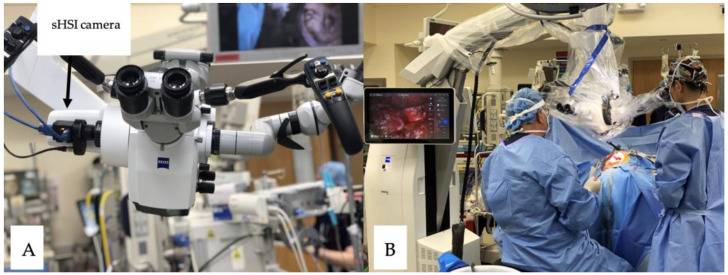
Surgical setup: (**A**) hyperspectral camera attached to operative (Zeiss surgical microscope) microscope; (**B**) HSI image visible on the screen during surgery.

Herein, we constructed a camera comprising a single housing unit with visible and infrared sensors linked via a beam splitter. A Bayer-like array with 16 visible (482, 493, 469, 462, 570, 581, 555, 543, 615, 622, 603, 592, 530, 540, 516, 503) and 25 infrared (613, 621, 605, 601, 685, 813, 825, 801, 789, 698, 764, 776, 750, 737, 712, 652, 660, 643, 635, 677, 865, 869, 854, 843, 668) wavelength (unit: nm) filters arranged in grid patterns was placed in front of each sensor. Images were collected from pediatric patients undergoing open brain surgery at the Children’s National Medical Center (IRB protocol number Pro00011028). Herein, subjects who were diagnosed with epilepsy or malignant neoplasm and planned to undergo surgical resection of pathological tissue were considered; moreover, they were required to be under the age of 18 and to provide consent for participation. The subjects were recruited from the physician’s pool of patients, and when they participated, a sHSI camera (BaySPec OCI™-D-2000 Ultra-Compact Hyperspectral Images) was attached to the operative microscope before the surgery (Figure 1A). During surgery, the staff captured periodic HSI and RGB images of the pathological brain matter (Figure 1B), which other study staff might classify. The images were then uploaded to a computer for processing.

In all of the cases, there was generally no interference in the circulatory conditions of the patients. We collected data from four patients: in three cases, a visible HSI camera was used; in two cases, RGB images were collected; and only in one case was an infrared HSI camera used during operation. 

### 2.2. Data Preprocessing

The images were collected using an RGB camera and two types of hyperspectral cameras: one in the visible spectrum and the other in the infrared spectrum; 136 RGB images, 279 visible hyperspectral images, and 85 infrared hyperspectral images were used. By eliminating images without the brain tissue, we obtained 60 RGB images, 234 visible HSI images, and 60 infrared HSI images. Visible HSI images were used to create a fourth dataset, which included only images with tumors (47 images). 

Using MATLAB (MATLAB 2023a, MathWorks NY USA), all hyperspectral images were separated based on wavelength, resulting in 16 images for each hyperspectral image in the visible spectrum and 25 for the infrared spectrum. This was performed to simplify the ground-truth segmentation and create a more detailed training dataset.

**Figure 2:**
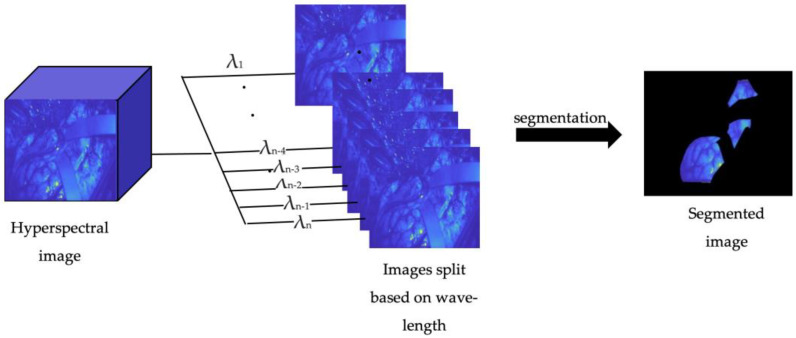
Preprocessing steps: original HSI image split based on wavelength and segmented.

Finally, using the ImageSegmenter tool in MATLAB, the ground truth segmentation was manually created by delineating and separating the healthy tissue from the background in the case of the first three datasets, as depicted in Figure 2. For the fourth dataset, the tumor and background were separated.

### 2.3. Machine Learning

Random forest is a machine learning model commonly used for classification and regression problems. It comprises multiple decision trees, which are used to obtain a single output. A decision tree is a type of machine learning algorithm that comprises nodes and branches, the nodes being decision points and the branches being possible outcomes. At each node, a decision is made to determine the branch to follow. The goal of a decision tree is to classify inputs into distinct categories. However, decision trees are prone to bias and overfitting. To avoid this issue, an ensemble of decision trees is used in the random forest algorithm [28]. 

**Figure 3:**
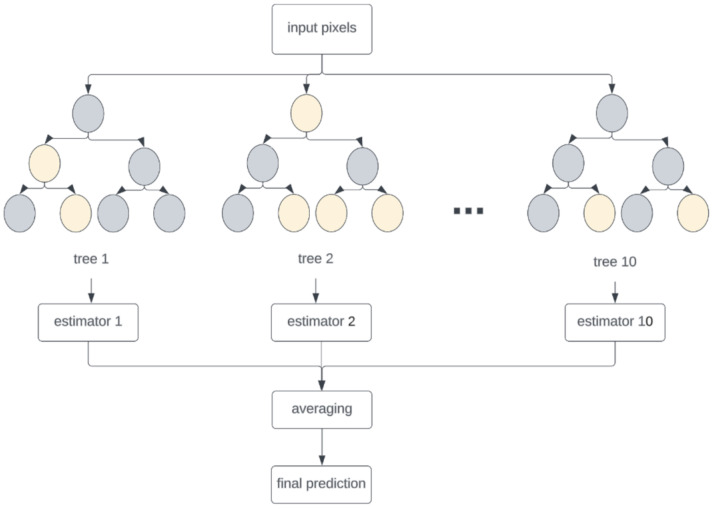
Random forest block diagram. Pixel variables can be divided into 0 (Gray circle) or 1 (Yellow circle).

We used random forest for the segmentation problem, as illustrated in Figure 3. This was performed by treating each pixel as a data point and assigning a label (0 for background and 1 for tissue). Random forest extracts features from an image via edge detection, pixel intensity evaluation, or texture analysis. Using these features and ground-truth labels, the random forest was trained by creating an ensemble of decision trees on a subset of randomly selected features. Once trained, each decision tree assigned a label to each pixel using majority voting, and a single label was generated for each pixel in the image. We selected random forest because of its superior capacity to handle large datasets, speed, and robustness to noise and outliers [29], which set it apart from other machine learning algorithms. 

Random forest with ten estimators was used to train our datasets. This was determined after comparing the average accuracy of the visible sHSI model for tissue segmentation at 2, 5, 10, and 15 estimators. The model was trained with each input column representing one of the wavelengths. For each model, the data were split in the ratio of 70:30 for training and testing, respectively.

### 2.4. Evaluation

To evaluate the model performance, we calculated the average intersection over union (IoU) and standard deviation.

$$\mathrm{IoU}=\frac{\mathrm{area}\;\mathrm{of}\;\mathrm{overlap}}{\mathrm{area}\;\mathrm{of}\;\mathrm{union}}.$$

Sensitivity and specificity were considered to evaluate the model’s performance.

### 2.5. Bench Top Testing

To assess the segmentation potential of random forest with an HSI camera, we initially tested it on a 24-colormap card; 43 images of the 24-colormap card were captured using the same HSI camera. The images were captured at different angles under various lighting conditions, and images with other objects next to or partially on top of the card were captured as well. By applying the same steps, the images were preprocessed and trained using random forest. Training was performed twice: initially on 30 random images (test 1) and then on 40 images (test 2); the remaining 13 and 3 images were used for testing. The average IoU value was considered to evaluate the model performance.

Furthermore, we compared random forest with another machine learning classifier, SVM. This was performed by calculating the average accuracy of each model trained on test 2.

## 3. Results

As summarized in Table 1, the average IoU values of the colormap card images ranged between 0.71 and 0.54 for tests 2 and 1, respectively, and the standard deviations were 0.1 and 0.01 for tests 1 and 2, respectively. Additional training data significantly improved the model’s performance (by approximately 20%). This case was considered while training the brain images.

**Figure 4:**
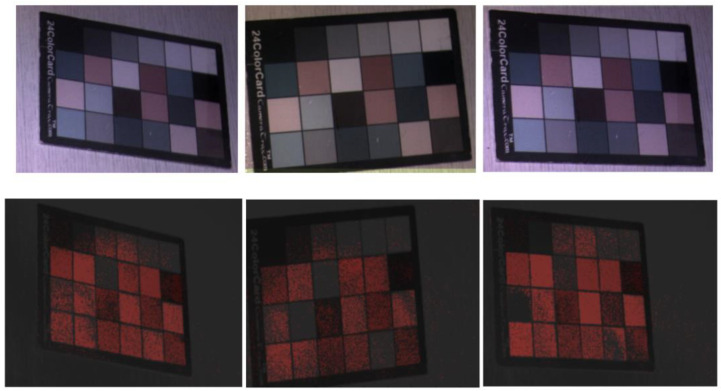
The 24-colormap captured using the hyperspectral camera. The three images on the top represent the 24-colormap at different angles where RGB images are false-colored for better visibility. The bottom three images represent the original hyperspectral black and white images overlayed with the segmentation results in red.

Figure 4 shows the 24-colormap images in black and white overlaid with segmentation resulting from random forest. As shown in the images, almost every small box was segmented, the lines between the boxes were always clearly black, and no background areas were segmented. Therefore, although some boxes were not entirely segmented, the model could distinguish the background from the region of interest. 

As one can see in Table 2, comparing random forest with SVM, the average accuracy of the random forest model was higher by 0.07. Furthermore, random forest is less computationally expensive and less likely to overfit due to noise. Since sHSI images are low-resolution images, random forest is the better choice. 

Table 3 shows that the average accuracy of the model increased by 0.01 between 2, 5, and 10 estimators. This value peaked at 10 estimators with an average accuracy of 0.854. Between 10 and 15 estimators, the average accuracy stayed stagnant. However, it was more computationally expensive and time consuming to train on 15 estimators. This is why the segmentation models were trained on 10 estimators. 

Table 4 lists the average IoU achieved for each of the four datasets. The highest average IoU (0.76) was achieved when the tissue was segmented using RGB images, followed by tissue segmentation using infrared HSI (0.59) and visible HSI (0.57). Finally, the lowest average IoU was achieved (0.10) when the tumor was segmented using visible HSI. The performance results of the visible and infrared HSI were compared; however, the visible HSI used images of three patients as opposed to one for the infrared image, which indicated that despite visible HSI producing slightly lower values, the segmentation was more robust. Furthermore, contrary to our initial predictions, RGB segmentation outperformed the other models.

Table 5 lists the average specificity and sensitivity values obtained by testing the models. The RGB model produced the highest sensitivity score (0.81). The second-highest sensitivity score was 0.50 for the visible HIS, followed by infrared HSI (0.45) and tumor segmentation (0.09). However, the opposite trend was observed for specificity. Tumor segmentation exhibited the highest specificity (0.996), followed by infrared HSI (0.93) and visible HSI (0.91). The lowest score was 0.72 for the RGB images. All the models exhibited high specificity values, which was not the case for sensitivity, specifically in the case of tumor segmentation. This is most likely due to the small region of interest that the tumor occupies compared to healthy brain tissue.

**Figure 5:**
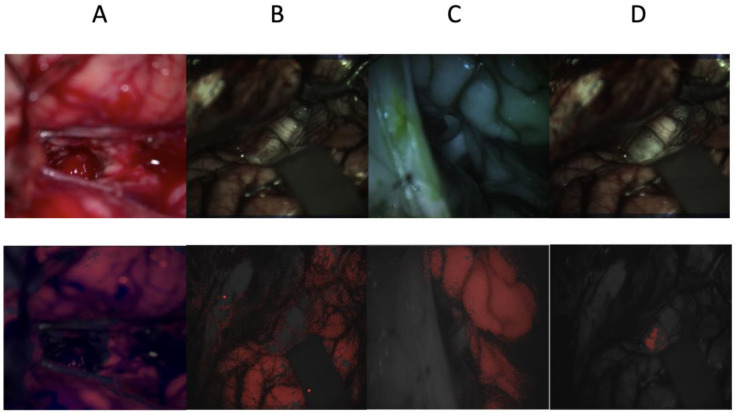
Pediatric brain images were captured using RGB, visible, and infrared HSI cameras. The four images at the top are the original RGB and hyperspectral images that have been artificially colored. The bottom four images are their respective segmentations overlayed in red. Images (**A**–**C**) are collected from the RGB, visible HSI, and infrared HSI datasets, respectively, where the healthy tissue is being segmented, and image (**D**) is obtained from the visible HSI dataset but for tumor segmentation.

Figure 5 shows each dataset, where the top image represents either the original RGB image of the hyperspectral image that has been artificially colored, and the bottom images represent the same images in black and white overlaid in red via segmentation. Image A indicates the high performance of the RGB model. The tumor was extracted from the unsegmented central area, and the surrounding tissue was healthy. The model correctly segmented the area of interest, which could be explained by the high sensitivity and specificity scores. Image B represents a visible HSI dataset of segmented healthy tissues. As shown in the image, random forest successfully excluded the surgical tools and skull; however, the margin of the healthy tissue was not clear. A similar observation can be made for image C (infrared HSI); however, it was less apparent, and the healthy tissue was more consistently segmented. Finally, in image D, which shows the visible HSI segmenting the tumor, the segmented area represents the tumor only; however, not all tumors were segmented. This was further demonstrated based on the high average specificity of 0.996. 

## 4. Discussion

The highest overall average IoU was achieved using the RGB images, with an average IoU of 0.76, and the HSI tissue segmentation models performed at average IoUs of 0.59 and 0.57, respectively. These results were significantly higher than those of tumor segmentation, which only achieved an average IoU of 0.10. However, the tumor segmentation achieved the highest average specificity of 0.996; when analyzing this and the example image, we can see that the model did not confuse the background information with the tumor; however, the lower sensitivity score indicates that the model had issues segmenting the entire tumor. The specificity scores of the other models were also very high, ranging from 0.93 to 0.72, indicating that the models can distinguish the background; however, the moderate sensitivity scores of the HSI models indicate that the overall tissue was not being segmented, which is a problem, specifically around the margins. Finally, the RGB model yielded a higher sensitivity score of 0.81. This model achieved significant results for all the metrics. 

Lean et al. [30] used a hyperspectral camera to collect images of patients with brain tumors who underwent brain surgery. The HSI spectra were in the visible and infrared regions, similar to those in our dataset. They segmented normal brain tissue and blood vessels using these images and machine learning classifiers. They used unsupervised and supervised machine learning algorithms, which were based on random forest. To train the models, they used images of the visible and infrared spectra and the fusion of the two images. Random forest achieved an accuracy of up to 93.10% and 82.93% for the infrared and visible spectra, respectively. 

Compared to our model’s performance, they were able to achieve significantly better results [30]; however, we were unable to compare dataset sizes owing to limited information. We assume that the difference in performance can be attributed to the aforementioned scenario. This motivates us to study a larger dataset to achieve better results in the future. 

## 5. Conclusions

In conclusion, our initial findings show great promise as we achieved an average IoU of 0.76 for the RGB dataset and 0.59 or 0.57 for the HSI datasets in the segmentation of healthy tissues. While the average IoU for tumor segmentation was lower at 0.10, however the specificity score of 0.996 provides strong evidence that the background segmentation remained accurate. Moreover, the high specificity observed in other models underscores the consistent segmentation of the region of interest. Notably, our models encountered challenges when segmenting tumor margins, an issue we aim to address through dataset expansion. However, the limitation of training and testing on a limited number of patients deserves consideration, as variations in tumor size and location across patients may impact model performance. This emphasizes the critical need for robust data collection and algorithm development. Additionally, the difficulty in visualizing non-surface brain tumors presents an obstacle, which we plan to overcome by integrating our sHSI camera with a laparoscope to capture multi-angle data. Future endeavors include developing a model capable of distinguishing between different brain regions (healthy tissue, tumor, and skull) and training a deep learning model with an expanded dataset.

## Figures and Tables

**Table 1:** Segmentation performance for the bench top test.

	Average IoU	Standard Deviation
Test 1	0.54	0.1
Test 2	0.71	0.01

**Table 2:** Average accuracy result of different machine learning classifiers.

	Average Accuracy
Random forest	0.84
SVM	0.77

**Table 3:** Average accuracy score of visible HSI model trained with different numbers of estimators.

Number of Estimators	Average Accuracy
2	0.834
5	0.844
10	0.854
15	0.854

**Table 4:** Segmentation performance of the four datasets using IoU.

	Average IoU	Standard Deviation
Tissue—RGB images	0.76	0.10
Tissue—Visible HSI	0.57	0.16
Tissue—Infrared HSI	0.59	0.20
Tumor—Visible HSI	0.10	0.09

**Table 5:** Average specificity and sensitivity of each dataset.

	Specificity	Sensitivity
Tissue—RGB images	0.72	0.81
Tissue—Visible HSI	0.91	0.50
Tissue—Infrared HSI	0.93	0.45
Tumor—Visible HSI	0.996	0.09

## Data Availability

Data is unavailable due to privacy or ethical restrictions.
